# Medical Applications and Cellular Mechanisms of Action of Carboxymethyl Chitosan Hydrogels

**DOI:** 10.3390/molecules29184360

**Published:** 2024-09-13

**Authors:** Weronika Kruczkowska, Karol Kamil Kłosiński, Katarzyna Helena Grabowska, Julia Gałęziewska, Piotr Gromek, Mateusz Kciuk, Żaneta Kałuzińska-Kołat, Damian Kołat, Radosław A. Wach

**Affiliations:** 1 Department of Biomedicine and Experimental Surgery, Faculty of Medicine, Medical University of Lodz, Narutowicza 60, 90-136 Lodz, Poland; weronika.kruczkowska@stud.umed.lodz.pl (W.K.); katarzyna.grabowska1@stud.umed.lodz.pl (K.H.G.); julia.galeziewska@stud.umed.lodz.pl (J.G.); piotr.gromek@stud.umed.lodz.pl (P.G.); zaneta.kaluzinska@umed.lodz.pl (Ż.K.-K.); damian.kolat@umed.lodz.pl (D.K.); 2 Department of Molecular Biotechnology and Genetics, University of Lodz, Banacha 12/16, 90-237 Lodz, Poland; mateusz.kciuk@biol.uni.lodz.pl; 3 Department of Functional Genomics, Faculty of Medicine, Medical University of Lodz, Zeligowskiego 7/9, 90-752 Lodz, Poland; 4 Institute of Applied Radiation Chemistry, Faculty of Chemistry, Lodz University of Technology, Wroblewskiego 15, 93-590 Lodz, Poland

**Keywords:** antimicrobial hydrogels, polysaccharide hydrogels, chitosan derivatives, biomaterials, cellular mechanism

## Abstract

Carboxymethyl chitosan (CMCS) hydrogels have been investigated in biomedical research because of their versatile properties that make them suitable for various medical applications. Key properties that are especially valuable for biomedical use include biocompatibility, tailored solid-like mechanical characteristics, biodegradability, antibacterial activity, moisture retention, and pH stimuli-sensitive swelling. These features offer advantages such as enhanced healing, promotion of granulation tissue formation, and facilitation of neutrophil migration. As a result, CMCS hydrogels are favorable materials for applications in biopharmaceuticals, drug delivery systems, wound healing, tissue engineering, and more. Understanding the interactions between CMCS hydrogels and biological systems, with a focus on their influence on cellular behavior, is crucial for leveraging their versatility. Because of the constantly growing interest in chitosan and its derivative hydrogels in biomedical research and applications, the present review aims to provide updated insights into the potential medical applications of CMCS based on recent findings. Additionally, we comprehensively elucidated the cellular mechanisms underlying the actions of these hydrogels in medical settings. In summary, this paper recapitulates valuable data gathered from the current literature, offering perspectives for further development and utilization of carboxymethyl hydrogels in various medical contexts.

## 1. Introduction

The medical sector is continuously seeking new ideas to improve treatment procedures, diagnostics, and patient comfort. Scientists are increasingly turning to nano, natural, composite, or other types of advanced biomaterials as solutions to satisfy these needs. These include hydrogels, which have been studied and used since 1960 [1]. The main reason why hydrogels arouse so much interest is their versatile properties and thus broad use due to the possibility of tuning their characteristics to fulfill the requirements of specific applications [2].

Hydrogels are materials utilized in biomedicine and biotechnology. Their features are specific networks made out of polymers connected by covalent or noncovalent bonds, weak interactions, and a significant amount of water filling the network. Therefore, hydrogels are capable of presenting both solid and liquid components (of, for instance, soft tissue), typically maintaining a uniform structure when a liquid or mechanical impact occurs [3,4]. Their structure and characteristics, namely, three-dimensionality (3D), flexibility, high water content, and ability to mimic the extracellular matrix (ECM) environment, make them applicable in various biomedical and clinical fields. Most of the medical implications are drug delivery via oral, subcutaneous, or topical route [5,6], tissue engineering (TE) regeneration and repair [7], wound and disease treatment [8,9,10], pain management [11], and antimicrobial application [12,13]. Additionally, hydrogels can be used in food industries as fat replacers or biosensors to identify risk factors [14].

Hydrogels can be made both with synthetic polymers, e.g., poly(ethylene oxide), poly(vinyl alcohol), and poly(ethylene glycol) [15], and natural polymers (e.g., chitin and its derivatives, agarose, alginate, agar, carrageenan) [16]. Special interest in the biomedical field was placed on chitosan hydrogels because of the biological activity of this polysaccharide. Chitosan (CS) is obtained by the deacetylation of its precursor, i.e., chitin (C_8_H_13_O_5_N)_n_ [17]. The acetyl groups (-COCH_3_) in the chitin molecule are eliminated during the deacetylation process, usually by alkaline conditions or enzymatic processes. Although there are differences in the degree of deacetylation, chitosan is usually thought to have at least 50% of its glucosamine units deacetylated. The molecule’s characteristics are altered by this procedure, increasing its solubility in acidic aqueous solutions and boosting its reactivity for further alterations [18,19]. Chitin is commonly found in species including arthropods, mainly crustaceans and insects, but it is also found in fungi. It is also one of the most common polysaccharide polymers in nature, occurring in the form of β-(1–4)-poly-*N-*acetyl-d-glucosamine [17,20]. Despite their broad application possibilities, chitosan hydrogels face certain restrictions, such as insolubility at pH > 6.0, that is, in physiological conditions, quick water adsorption, and relatively poor mechanical properties [21,22]. To improve their performance and overcome the above-mentioned limitations, chitosan hydrogels, or, more precisely, chitosan macromolecules, are subject to modifications. One alteration is carboxymethylation. This reaction is based on the substitution of carboxymethyl groups (-CH_2_-COOH) into chitosan molecules, typically through monochloroacetic acid in alkaline conditions [23,24]. The carboxymethyl chitosan (CMCS) obtained in this reaction is also biocompatible and antimicrobial, but it presents added value as compared with chitosan, i.e., solubility in a broader range of pH values. Therefore, hydrogels based on CMCS may be of similar use as chitosan hydrogels, but they are advantageous in terms of their manufacturing [25,26].

Considering cellular mechanisms, carboxymethyl chitosan and its hydrogels may have an enhanced impact on cell behavior and cellular signaling compared with unmodified chitosan [27]. This review focuses on carboxymethyl chitosan hydrogels’ influence on cellular mechanisms in biomedical applications. Aspects of how hydrogels can affect angiogenesis, the action and functioning of fibroblasts, and collagen deposition are considered. It also focuses on microbiological issues and the potential applications of CMCS hydrogels in terms of their properties and mechanisms influencing antibacterial and antimicrobial reactions, as well as the immunology context of the induction of proteins involved in the immune system response and inflammation.

## 2. Manufacturing and Structure of Carboxymethyl Chitosan Hydrogels

Hydrogels are three-dimensional networks primarily composed of hydrophilic polymers that possess the unique ability to absorb and retain significant amounts of water within their structures. Typical synthetic polymers suitable for hydrogel manufacturing include poly(hydroxyethyl methacrylate) (PHEMA), polyethylene glycol (PEG), polyacrylic acid (PAA), and poly(vinyl pyrrolidone), among others [15,28,29,30]. In contrast, natural-based polysaccharides of interest include alginate, elastin, cellulose derivatives, pectin, and chitosan [16,31,32,33]. Chitosan, in particular, has garnered significant attention in recent research because of its ease of manufacture and modification, as well as the biological activity of its natural components. The synthesis of hydrogels, regardless of their base materials, typically involves cross-linking hydrophilic or polymeric chains. This cross-linking process can simultaneously occur with polymerization, resulting in a three-dimensional network capable of absorbing water. This network can be modified in various ways, granting specific types of hydrogels their distinct features.

**Figure 1:**
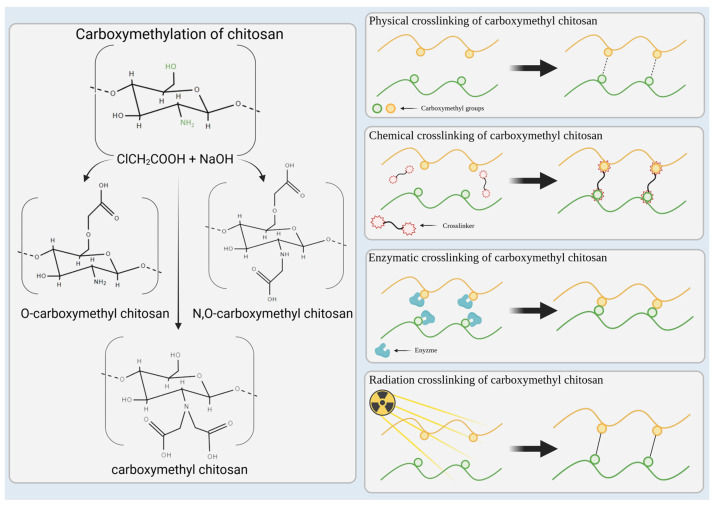
Carboxymethylation and cross-linking methods of chitosan. Carboxymethylation of chitosan is a chemical modification process that produces different derivatives of chitosan. The reaction with ClCH_2_COOH and NaOH leads to two products, i.e., *O-*carboxymethyl chitosan and *N*,*O-*carboxymethyl chitosan. There are also different types of cross-linking that carboxymethyl chitosan can undergo including physical, chemical, enzymatic, and radiation cross-linking. Each cross-linking method forms connections among the polymer chains, chemical bonds, or interactions based on affinity.

Chitosan has emerged as a fundamental material for hydrogels in recent decades. It is a cationic natural polysaccharide derived from chitin [10,26]. The structure of chitosan consists of two types of units, i.e., the deacetylated unit, d-glucosamine, and the acetylated unit, *N-*acetyl-d-glucosamine, which are randomly distributed and connected by β-(1–4) bonds, as illustrated in Figure 1 [34]. Chitosan is characterized by its excellent biocompatibility and biodegradability, and it can be converted into gels through methods such as chemical cross-linking or freeze–thawing [35]. These properties make chitosan an ideal candidate for active hydrogels. However, a significant drawback of chitosan is its limited solubility in water, particularly at physiological pH, as well as in most organic solvents [36,37]. Fortunately, the presence of C6-OH, C3-OH, and C2-NH_2_ groups in chitosan allows for its modification. This modification involves introducing new functional groups, leading to the development of advanced forms of chitosan, such as Schiff-alkalized, PEGylated, or carboxymethyl chitosan (Figure 1) [26,34,36,38].

Carboxymethyl chitosan stands out as a promising derivative because of its superior solubility and reduced degradation compared with its parent, chitosan. Additionally, CMCS exhibits an amphoteric character and can be synthesized through a relatively straightforward process [26,38]. The carboxymethylation process involves the reaction of chitosan with monochloroacetic acid in an alkaline medium. Sodium hydroxide facilitates this reaction by protonating the amino and hydroxyl groups in chitosan, while monochloroacetic acid serves as the carboxymethylation agent. The resulting carboxymethyl chitosan (CMCS) can then be dried to produce a powdered form. The entire manufacturing process for powdered carboxymethyl chitosan takes approximately 48 h, which includes a 24 h drying period [23,39]. Once the powdered CMCS is dissolved, cross-linking of its macromolecules can be performed using various methods.

Cross-linking is a critical process for modifying both synthetic and natural-based polymers to create durable hydrogels. Cross-linking methods can be categorized into several types including physical, chemical, radiation, and enzyme-mediated cross-linking [29,40]. Physical cross-linking is reversible and does not utilize multifunctional cross-linkers. It relies on the formation of physical interactions among the functional groups of polymers, which prevent the dissolution of the hydrogel. Physical cross-linking includes ionic and hydrophobic interactions, hydrogen bonding, and complex coacervation [41]. Chemical cross-linking results in covalent bonding among macromolecules, leading to permanent links. It employs cross-linkers to combine their functional groups (principally unsaturated bonds, but also amino, carboxyl, and alcoholic) with the polymer chain. Various techniques for chemical cross-linking include free radical polymerization, Schiff base reactions, polymer–polymer conjugation, and chemical reactions of complementary groups [42]. Radiation cross-linking typically does not require chemical additives and allows for simultaneous alteration and sterilization, making it particularly convenient for certain applications. This process involves exposing the polymers in solution to different radiation, such as gamma rays, electron beams, or X-rays. The outcome of radiation-induced cross-linking is a permanent hydrogel, which can be a cost-effective solution for hydrogel fabrication [43,44]. Enzymatic cross-linking is a novel method that utilizes enzymatic reactions to create covalent bonds among polymers, which may eventually form a hydrogel. Various enzymes, including tyrosinase, phosphopantetheine transferase, lysyl oxidase, plasma amine oxidase, and phosphatases, can be employed. Each enzyme operates through different mechanisms but ultimately leads to the synthesis of durable hydrogels [29]. The cross-linking methods mentioned above are illustrated in Figure 1.

The incorporation of –CH_2_COOH carboxymethyl groups into the polymer structure typically increases viscosity, enlarges the hydrodynamic volume, reduces toxicity, and enhances the biocompatibility of the polymer [45]. The addition of carboxymethyl groups into the chitosan structure still allows for the formation of gels based on ionic interactions or hydrogen bonding. However, these constructs may be unstable; therefore, to ensure the stability of the hydrogel, chemical cross-linking is often necessary. This can be achieved through the various cross-linking techniques mentioned earlier, such as employing poly(ethylene glycol) diacrylate (PEGDA) or epigallocatechin-3-*O-*gallate (EGCG) as cross-linking agents (Figure 1) [46,47]. Among the cross-linking strategies, radiation-induced cross-linking appears to be particularly effective. By utilizing ionizing radiation, and sometimes requiring the addition of a suitable cross-linker, it is possible to create a durable hydrogel that is also sterile when a specific radiation dose is applied. This provides a hydrogel suitable for various medical applications, including wound healing, as well as serving as an antimicrobial and antifungal active material or for drug delivery [21,48]. The resulting hydrogel is characterized by its safety, durability, biocompatibility, and biodegradability CMCS can also be cross-linked in a variety of ways, each of which can affect the final characteristics of the hydrogel. These methods include physical (e.g., ionic interactions), chemical (e.g., covalent bonding), radiation-induced, and enzymatic approaches. The three-dimensional network that makes up the structure of CMCS hydrogels is able to absorb and hold vast amounts of water, and the carboxymethyl groups give the hydrogel an amphoteric quality that enables it to react to pH changes. A distinct swelling profile is produced by this pH responsiveness; there is moderate swelling at low pH values, deswelling at pH values of about 3–5, and extensive swelling at higher pH values. Those are governed by the ionization of ionogenic groups of amines and carboxylic. By modifying variables like the degree of carboxymethylation, cross-linking density, and addition of additives, the synthesis process and structural properties of CMCS hydrogels can be customized to meet particular biomedical applications, such as drug delivery, tissue engineering, and wound healing.

## 3. Biochemical Properties of Carboxymethyl Chitosan Hydrogels and Their Potential Applications

**Figure 2:**
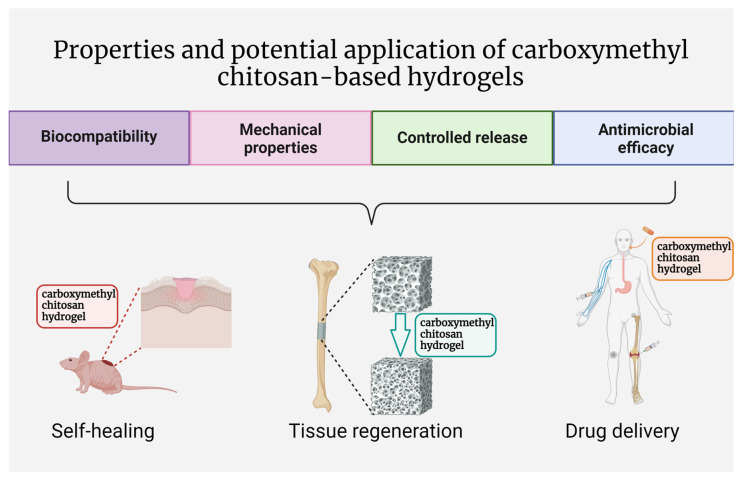
Properties and potential application of carboxymethyl chitosan-based hydrogels. CMCS hydrogels are known for their vast properties and application, with key examples depicted in the figure. Biocompatibility makes hydrogels applicable in the medical field, while their porous structure allows for the incorporation of drugs or bioactive molecules into their structure. This, combined with potential biodegradation, enables the controlled release of these substances. Hydrogels can also provide antimicrobial properties, which is attractive in medical applications. The major applications include wound healing, tissue regeneration, and targeted drug delivery from the hydrogel carrier.

Carboxymethyl chitosan hydrogels have emerged as a versatile class of biomaterials with unique biochemical properties, offering a wide range of potential applications in various fields, especially in biomedicine. The CM-modified chitosan polysaccharide possesses enhanced solubility, and the hydrogels thereof are biocompatible with tailored mechanical strength, degradation kinetics, or evidenced antibacterial properties [18,21,26]. These features combined enable them to suit specific requirements, rendering them highly adaptable for use in drug delivery, tissue engineering, wound healing, and beyond [49,50,51]. This section explores the biochemical properties of carboxymethyl chitosan hydrogels and highlights their promising potential in various biomedical applications, which are also illustrated in Figure 2.

### 3.1. Biocompatibility

Implementing natural polymers as base materials in hydrogels provides biocompatibility and biodegradability [52,53,54]. In a recent paper, Kłosiński et al. assessed the biocompatibility of CMCS hydrogels. Their results indicated no adverse effects in the tissue, confirmed the said property, and suggested their potential use as a wound-healing dressing in the future. Their study design involved a PEGDA cross-linking agent to form CMCS hydrogels via the radiation method [26]. The CMCS hydrogel accelerated the healing of a chronic wound model in rats over two weeks and promoted wound closure and tissue regeneration [10,55]. Another study focusing on burn wound healing evaluated carboxymethyl chitosan hydrogel formulation combined with hyaluronic acid (HA) and silver. The methodology involved the analysis of morphometric, macroscopic, and microscopic aspects and collagen quantification. The CMCS hydrogel treatment significantly impacted wound contraction, granulation tissue formation, inflammatory infiltration, and collagen fiber deposit during the burn wound healing phases. It was shown that the group treated with CMC had greater collagen deposition (around 15 on the scale) than the groups treated with CMC/HA and CMC/Ag (around 6–7 on the scale) [23]. Kabirkoohian et al. focused on the tissue engineering aspect of carboxymethyl chitosan chemically immobilized on polycaprolactone nanofibers to create scaffolds for osteochondral tissue engineering applications. The design concept of CMC-immobilized scaffolds aimed to create a biomimetic environment resembling the natural extracellular matrix (ECM) found in osteochondral tissue. Carboxymethyl chitosan was chosen because of its biocompatibility, biodegradability, and structural similarity to glycosaminoglycans (GAGs), which are crucial components of the cartilage ECM. The scaffolds showed good biocompatibility with cell viability of over 82% and supported human bone marrow mesenchymal stem cells (hBM-MSCs). The scaffolds were created through chemical immobilization, enhancing their bioactivity and mechanical properties. The scaffold architecture supported cell attachment and proliferation, with uniform CMC distribution facilitating cell–matrix interactions. The scaffolds induced diverse osteochondral differentiation pathways without external agents and promoted calcium phosphate (CaPO_4_) or hydroxyapatite formation, essential for osteogenesis. Strong cell–matrix interactions were crucial for the scaffold’s mechanical stability and functional integration. Higher CMCS content presented more chondro-inductivity [56].

Carboxymethyl chitosan-based hydrogels’ adaptability also allows for modifications such as the addition of aldehyde groups, for example, polyethylene glycol (PEG) or four-armed PEG. These modifications impart self-healing properties, which hold significant potential for applications in wound healing, tissue regeneration, and drug delivery [57]. Huang et al. crosslinked CMCS with benzaldehyde-terminated telechelic four-armed polyethylene glycol (PEG-BA) and investigated its self-healing properties. The hydrogel had a good storage modulus and self-healing behavior (after rupture, the gel could return to its state before rupture) and showed cytocompatibility. Moreover, it emerged as a potential novel hemostatic material, since it effectively stopped bleeding when injected into a rabbit liver, reducing total blood loss and hemostasis time [58]. Another possible alteration is combining CMCS with other materials, such as polyvinyl alcohol. Liu and coworkers loaded this type of hydrogel with silver nanoparticles, rendering antibacterial and, more importantly, self-healing properties. The latter was achieved by double cross-linking borate ester bonds. Combining those two properties with non-toxicity allowed for its practical application in wound dressings [59]. Yet another modification of CMCS is thiolation, which greatly improves carboxymethyl chitosan mucoadhesive, permeation, and other functional qualities [60]. A study published in 2020 presented thiolated carboxymethyl chitosan-based 3D scaffolds and their evaluation for skin repair and tissue regeneration. The scaffolds had a highly porous structure and adequate mechanical properties for skin repair, as well as good in vitro biocompatibility and other physicochemical characteristics appropriate for tissue regeneration [61]. Therefore, the biocompatibility of CMCS hydrogels, along with their adaptability to various modifications, makes them highly promising materials for a wide range of biomedical applications, including wound healing, tissue engineering, and drug delivery.

However, hydrogels frequently contain traces of dangerous cross-linker residues that can contaminate food and impose safety concerns. To prevent toxicity from cross-linker residues in hydrogels, several strategies can be employed. These include using natural cross-linkers (e.g., genipin, tannic acid), developing cross-linker-free hydrogels, designing controlled release mechanisms, employing bio-orthogonal chemistry, utilizing physical cross-linking methods (e.g., hydrogen bonding, ionic interactions), and incorporating plasticizers. These approaches aim to maintain hydrogel functionality while enhancing safety for food and biomedical applications, reducing the risk of toxic residues without compromising the desired properties of the hydrogels [42,62,63,64]

### 3.2. Mechanical Properties

Carboxymethyl chitosan hydrogels exhibit a range of mechanical properties influenced by the molecular weight and degree of carboxymethylation of the polymer, cross-linking density, and the presence of additional components. These properties can be tailored through various modifications to suit specific applications. In a previous study, Kłosiński et al. demonstrated that CMCS hydrogels with a high degree of carboxymethyl substitution (96%), when cross-linked with PEGDA using a radiation method, displayed effective fluid absorption and retention capacities. The process consisted of irradiating at room temperature using an electron beam (EB) to an overall dosage of 25 kGy. The addition of PEGDA significantly improved gel formation efficiency, wet dimensional stability, and tensile properties compared with CMCS gels without the cross-linker, which were manufactured also by radiation processing [26]. Hu et al. enhanced the mechanical properties of CMCS hydrogels by incorporating zinc acetate as a catalyst and polyvinyl alcohol (PVA) into the composite. Different formulations of CMCS and PVA were anticipated to create a hydrogel with the desired gelation time and stability, and PEGDA 500 was used as a cross-linker. The hydrogel containing 1% PVA resulted in a good compressive strength (1.96 MPa) and a high water absorption capability of 1860%. The hydrogels display pH-sensitive swelling behavior from 3 to 11 because of ionogenic carboxyl and amine groups [65]. Razmjooee et al. created a triple-network hydrogel combining physically cross-linked gelatin with covalently cross-linked polyacrylamide and CMCS. By adjusting the concentration of the cross-linking agent, N,N’-methylene bisacrylamide, they achieved tailored porosity, good mechanical strength, and a high swelling ratio [66]. Feng et al. developed a potential wound dressing by incorporating tannic acid (TA) into a PVA-CMCS composite hydrogel. The addition of TA enhanced the compressive strength (311.9 ± 9.5 kPa to 507.2 ± 3.5 kPa) and stiffness (2.6 ± 0.5 MPa to 4.2 ± 0.2 MPa) of the structure, although the additive slightly reduced swelling potential [67]. Mo et al. further improved CMCS hydrogel properties by double-cross-linking with silane and Ca^2+^ and adding silver nanoparticles. This modification resulted in enhanced stretchability, toughness, and tissue adhesion, making it suitable for applications such as wound healing [68]. The wide range of mechanical properties of carboxymethyl chitosan hydrogels, and the ability to influence them in various ways, i.e., adding different components such as PVA or TA, can benefit their application in numerous fields, like wound dressing and healing.

#### Tailored Solid-State Properties

CMCS hydrogels can be engineered to exhibit specific properties suitable for various applications. CMCS hydrogels are inherently biodegradable because of their natural polymer base. The rate of degradation can be controlled by adjusting the degree of carboxymethylation and cross-linking density.

As demonstrated by Feng et al., the incorporation of components like tannic acid can significantly enhance the antibacterial properties of CMCS hydrogels [67]. Additionally, the integration of silver nanoparticles, as shown by Mo et al., can further boost antimicrobial efficacy [69]. The high water absorption capability of CMCS hydrogels, particularly evident in Hu et al.’s work (1860% water absorption), makes them excellent moisture-retaining materials, beneficial for applications like wound dressing. CMCS hydrogels exhibit pH-responsive behavior because of their ionogenic carboxyl and amine groups. Hu et al. observed pH-sensitive swelling behavior in the range of pH 3 to 11 [65]. This property allows for the controlled release of active ingredients or selective absorption in specific pH environments.

By manipulating these properties through various modifications and additives, researchers can design CMCS hydrogels tailored for specific applications in fields such as wound healing, drug delivery, and tissue engineering. The versatility of CMCS hydrogels in achieving desired mechanical and functional properties makes them a promising material for ongoing research and development in biomedical applications.

### 3.3. Controlled Release

Carboxymethyl chitosan has ionogenic functional groups, gel-forming ability, and low toxicity, as well as good biodegradability and ease of modification. All these aspects make CMCS, and, by extension, its hydrogels, an excellent potential carrier for the controlled release of drugs, vitamins, etc., into the human organism [25].

As stated by Bai et al., CMCS-based nanoparticles (NPs) have been studied for their potential in the delivery of genes and antigens, therapeutic proteins, and anti-cancer agents. Furthermore, the researchers investigated the optimization of the nanoparticle fabrication method. These NPs were intended for use in the sustained release of insulin. Using the ionic gelation method, pH-responsive nanoparticles of carboxymethyl chitosan were created with Ca^2+^ ions. CMCS was dissolved at various concentrations, whereas insulin was dissolved in a hydrochloric acid solution (pH 2). A total of 1 mL of insulin solution was added dropwise to 8 mL of a CMCS solution while stirring constantly. Following that, 1 mL of CaCl_2_ solution was added to the mixture dropwise and stirred continuously for 1 h until NPs were formed. The development of insulin loading using various fabrication factors such as protein quantity, CMCS concentration, and cross-linking density was followed by the examination of insulin release in vitro. The team reported that the modifications to the cross-linking density offered an easy method for altering the characteristics of the NPs to differ in insulin concentrations in simulated conditions—obviously, the cumulative release of insulin reduced as the cross-linking density increased. These discoveries contributed to the development of a straightforward insulin-loading method and its controlled release [70]. One study implemented carboxymethyl chitosan–soy protein (CMSC/SPI) complex nanoparticles in order to release vitamin D3 (VD) in a controlled manner. Teng et al. demonstrated a remarkable encapsulation of the vitamin, the desired stability of the intricate nanoparticles, and a regulated release profile. To achieve that, nanoparticles were prepared in the dark using the ionic gelation method, and, as a control, blank nanoparticles were also prepared. The design concept involved creating complex nanoparticles from carboxymethyl chitosan and soy protein isolate using ionic gelation with Ca^2+^ to encapsulate vitamin D3, a hydrophobic micronutrient. This CMCS/SPI complex system required less Ca^2+^ than CMCS alone and formed stable particles over a broader pH range, resulting in higher loading and encapsulation efficiencies. The nanoparticles demonstrated reduced release in simulated gastric fluid and increased release in simulated intestinal conditions, making them promising for controlled delivery of hydrophobic nutraceuticals and bioactives [71]. Another application of CMCS-comprising delivery systems is treating inflammatory bowel disease (IBD). One significant disadvantage of oral therapy for IBD is the non-specific dispersing of medicines over time. Despite its anti-inflammatory properties, the poor bioavailability of curcumin limits its use in IBD therapy. To address this issue, Zhang and colleagues created a pH-sensitive combination hyaluronic acid/gelatin (HA/GE) micro-hydrogel incorporating CMCS microspheres loaded with curcumin as a drug delivery system. This multi-layered system protected the drug from degradation in the upper gastrointestinal tract, allowing targeted release in the colon where the hydrogel disintegrated because of the higher pH. The microspheres then provided sustained release of curcumin, enhancing its bioavailability and maintaining therapeutic levels in the colon for extended periods, potentially improving the efficacy of IBD treatment. The microsphere formulation could make medications more stable and, compared with formulations without microspheres, could enhance the hydrogel’s capacity to release molecules over time. The formulation demonstrated strong sustained release properties in vitro, with a drug release rate of 65% over 50 h. In vivo pharmacokinetic tests revealed that a high level of CUR was sustained in colon tissue for over 24 h. Additionally, the formulation significantly reduced levels of pro-inflammatory cytokines IL-6 and TNF-α compared with the control group. The trial results demonstrated the potential of the composite gel delivery technology in successfully administering CUR in the treatment of colitis [72]. The goal of Gholamali et al. was to create an advanced drug release system utilizing CMCS/starch with various weight ratios, incorporating CuO-NPs that would allow for controlling the release properties of the formulation, in this case, Amoxicillin. Starch’s low viscosity, transparency, swelling, and significant strength make it an effective food additive; it is also used in medical fields including drug delivery systems. It was found that the swelling capacity of nanocomposites was determined by the concentration of nanoparticles in the CMCS/starch hydrogels. Increasing the copper chloride concentration increased the swelling of the nanocomposite hydrogels, which in principle was pH-dependent. The CMCS/starch hydrogel with CuO nanoparticles exhibited longer and more regulated drug releases than those without it, and that control increased with the number of NPs added. The tighter and flatter surface of CMCS/starch nanocomposite hydrogels could prevent drug molecules from passing through, leading to this behavior. The combination of these findings also suggested that the CMCS/starch nanocomposite was a promising solution for controlled drug delivery [73]. In conclusion, because of its properties such as non-toxicity and ionic character, CMSC hydrogels are promising materials for controlled drug release. The examples above highlight the versatility of these hydrogels and the undemanding modifications of the aforementioned properties, which again allows scientists to achieve different patterns of controlled release for various drugs and make therapies more efficient.

### 3.4. Antimicrobial Efficacy

CMCS and its hydrogels are known for their antibacterial properties, which is connected to the presence of amino groups in the structure of chitosan, the parent polysaccharide [74]. One study incorporated fungal-derived carboxymethyl chitosan into bacterial cellulose (BC) in order to enhance the antibacterial properties of a hydrogel. Introducing CMCS into bacterial cellulose, i.e., bringing amino groups to the hydrogels renders the material’s ability to combat bacterial infections. The antibacterial activity of CMCS-BC hydrogels was evaluated against *Escherichia coli* and *Staphylococcus aureus*. Overall, the hydrogel physiochemical characteristics and antibacterial efficacy were improved by the impregnation of CMCS into BC fiber networks, which suggested that the hydrogel could be a good option for wound dressing applications that demand antibacterial qualities. This was also due to improved swelling, where CMCS-BS reached about 6000% water uptake whereas, pure BC was below 4000%. [74]. Their antibacterial properties may be of use in wound healing, endoscopic submucosal dissection, and resulting forms of iatrogenic ulcers. An injectable carboxymethyl chitosan hydrogel showed resistance to *S. aureus* and *E. coli*. It is also worth mentioning that the hydrogel exhibited other properties characteristic of such material, such as good film-forming, adhesion capabilities with pH sensitivity, and swelling rates, as well as suitable degradation [75]. Yu et al. also proposed injectable CMCS hydrogel for antibacterial and anti-tumor recurrence applications. They proved such abilities on a mouse model and an *S. aureus*-infected model of mice, respectively [76]. On the other hand, Zhang and coworkers prepared a high-mechanical strength carboxymethyl chitosan-based hydrogel film for antibacterial wound dressing. It exhibited high tensile strength (31.7 MPa) and elongation at break, good swelling behavior, and effective inhibition of bacterial growth. These results suggested its potential for use in wound dressing applications. According to the disk diffusion test results, it demonstrated strong antibacterial activity against *S. aureus* and *E. coli* [77]. Chaiwarit et al. used clindamycin HCl as a model drug loaded in CMCS Ca^2+^-cross-linked gel nanoparticles. Ultrasonication was used to produce nanoparticles of 543.63 ± 55.07 nm to 318.40 ± 7.56 nm diameter depending on the amplitude and time. The nanoparticles demonstrated extended-release and good antibacterial activity against *S. aureus* and *Cutibacterium acne*, suggesting ion-stabilized CMCS nanogels as a potential delivery system [78]. Ciprofloxacin (CIP) in the interconnected networks of Fe^3+^-induced self-assembled CMCS, used in this example, could also be implemented as a treatment in osteomyelitis (OM), which is known for being complex, aggressive, and heterogeneous. Therefore, OM makes it difficult to eradicate all germs and promote osteogenesis. By accelerating the early phases of infected bone healing, this injectable antibacterial and osteogenic-promoting hydrogel was deemed a highly promising platform for the low-cost, safe, and successful treatment of OM [79]. Another study took a different approach and focused on a bacteria belonging to family of *Streptococcaceae*—*Pediococcus pentosaceus*. It combined carboxymethyl chitosan with *N-*acetylneuraminic acid (C-NeuAc) to create probiotic suspension and encapsulate *P. pentosaceus*. The hydrogel passed through the digestive system with an 80% survival rate, demonstrating good biocompatibility and minimal cytotoxicity. It was discovered that freeze-drying and low temperatures were the best ways to guarantee long-term storage, which made the C-NeuAc hydrogel a promising food-grade probiotic delivery method [80].

CMCS hydrogels also exhibit antifungal properties. *Candida* is a fungus causing infections in immunocompromised people and, in general, when the natural microbiota is altered, with *C. albicans* being the most common human pathogen and causing both mucosal and deep-tissue infections [81,82]. Chitosan on its own provides certain antifungal properties, which are enhanced by chemical modifications, for instance, to carboxymethyl chitosan [83,84]. Highlights from a study published in 2018 focused on the antifungal activity of CMCS hydrogels on gauze. This study also paid attention to the fact that the hydrogels in question had better antifungal properties compared with gauze-coated chitosan and non-coated gauze. Kurniasih et al. also mentioned that because of their high water solubility, CMCS hydrogels are easier to apply than chitosan [83]. Another study focused on antifungal properties against *C. albicans*, *C. krusei*, and *C. glabrata*. Different concentrations of CMCS and chitosan were prepared, and their effects on yeast growth were measured using a microplate nephelometer over 24 h. The results were analyzed to determine the minimum inhibitory concentrations (MICs) and the correlation between substance concentration and antifungal activity. Carboxymethyl chitosan displayed a delay in the growth of *Candida* species, particularly noticeable at higher concentrations. However, its antifungal activity was found to be less potent compared with the derivatives of chitosan hydrochloride, which was highlighted by a small to moderate negative relation between carboxymethyl chitosan concentration and relative growth after 24 h (Spearman correlation coefficients: *C. albicans* −0.644, *C. krusei* −0.418, *C. glabrata* −0.360) [85]. The antimicrobial efficacy of CMCS and its hydrogels, both antibacterial and antifungal, makes them highly promising materials for various biomedical applications, including wound dressings, drug delivery systems, and food-grade probiotic delivery methods. The information of properties described above is summarized in Table 1.

### 3.5. Summary of the Biochemical Properties of Carboxymethyl Chitosan Hydrogels

Carboxymethyl chitosan hydrogels exhibit remarkable biochemical properties that make them highly suitable for various biomedical applications (Table 1). Biocompatibility, an essential characteristic of CMCS for biomedical use, was examined in many in vitro and in vivo studies. Properties like non-toxicity and boosted cell proliferation and differentiation confirm this finding. Many research groups confirmed that the biocompatibility of CMCS refers to many types of cells, including fibroblasts, stem cells, and others. Carboxymethyl chitosan hydrogel can be modified by adding PEGDA, aldehyde groups, polyvinyl alcohol, or other substances. These modifications can improve the hydrogel’s capacity for self-healing, wound healing, skin repair, tissue engineering, and interactions with cell viability. The performance of CMCS hydrogels in diverse biological applications is largely dependent on their mechanical properties. Modifications of carboxymethylation, cross-linking density, and addition of PEGDA, polyvinyl alcohol, or tannic acid can successfully improve fluid absorption, retention, wet dimensional stability, and tensile properties. Furthermore, the aforementioned mechanical properties enable CMCS to respond to external stimuli. As a result, such hydrogels offer applications in both wound healing and medication administration. CMCS hydrogels can be used as a matrix for different bioactive compounds with controlled release. The controlled release of medications, proteins (insulin), vitamins, and other bioactive substances (curcumin) has been demonstrated for CMCS hydrogels. The release kinetics can be modified by the hydrogel composition, cross-linking density, and environmental factors. Targeted drug delivery, tissue engineering, wound healing, and other therapeutic domains are only a few of the therapeutic fields in which CMCS hydrogels’ adaptability in controlled-release applications is demonstrated. A number of processes, including bacterial cell membrane breakage, bacterial growth suppression, and metal ion chelation, are thought to be responsible for the antimicrobial action of CMCS hydrogels. A variety of pathogens, including Gram-positive bacteria (like *S. aureus*), Gram-negative bacteria (like *E. coli*), and fungi (like *Candida* species), have been shown to be effectively combated with CMCS-based hydrogels. Other antimicrobial agents, including zinc ions or silver nanoparticles, can be added to further strengthen the antimicrobial characteristics. Because of these properties, CMCS hydrogels are particularly well-suited for usage in antimicrobial textiles, wound dressings, and other medical contexts where infection control is a challenge.

## 4. Cellular Mechanisms of Action of Carboxymethyl Chitosan Hydrogels

**Figure 3:**
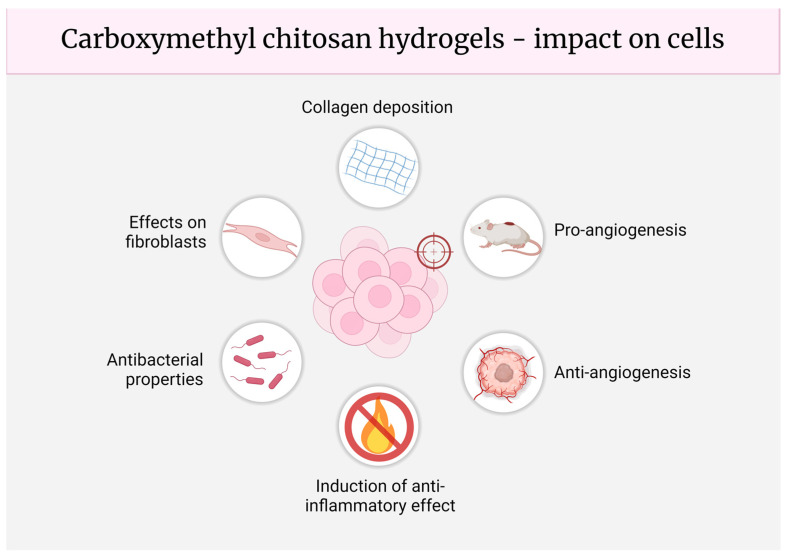
Carboxymethyl chitosan hydrogels—impact on cells. The cellular effects of carboxymethyl chitosan hydrogels include promoting collagen deposition, influencing fibroblasts, stimulating or inhibiting angiogenesis, providing antibacterial properties, and reducing inflammation. These diverse effects highlight the potential of these hydrogels in various biomedical applications, such as tissue engineering, wound healing, and disease treatment.

Carboxymethyl chitosan hydrogels have emerged as versatile biomaterials with significant potential in various biomedical applications. These hydrogels exhibit a wide range of beneficial biological properties, including antibacterial and anti-inflammatory activities, effects on angiogenesis and on fibroblasts, and the promotion of collagen deposition. Understanding the cellular mechanisms of action underlying these activities is crucial for developing and optimizing CMCS-based materials for specific therapeutic uses (Figure 3).

### 4.1. Angiogenesis

According to the National Library of Medicine, angiogenesis is the growth of new blood vessels from pre-existing vasculature, occurring throughout life in both health and disease. It occurs in all tissues and is regulated by oxygen and hemodynamic factors. Controlling angiogenesis has therapeutic value, with stimulation being beneficial in ischemic heart disease, peripheral arterial disease, and wound healing and inhibition being beneficial in cancer, ophthalmic conditions, and rheumatoid arthritis [90,91]. Carboxymethyl chitosan’s impact on angiogenesis is crucial since it may be of use in several applications. Firstly, by inhibiting angiogenesis in cancer, CMCS can be applied in order to restrict tumor growth and spread [92]. Secondly, its ability to promote angiogenesis may be useful in wound healing and tissue engineering, especially vascular tissues [23,93].

Regarding the inhibitory effect on angiogenesis, several mechanisms at the cellular level make carboxymethyl chitosan and its hydrogels effective. CMCS may repress tumor angiogenesis by directly inhibiting endothelial cell migration and downregulating pro-angiogenic factors [92]. First, human endothelial cells and their migration play a primary role in angiogenesis since during this process, they need to migrate to specific locations to create new vascular networks. This allows tissue growth and repair and is crucial for various physiological mechanisms, such as embryonic development, wound healing, and tissue regeneration. There are different subtypes of endothelial cells including arterial, capillary, and venous. The last ones have been identified as key sources of neo-angiogenic endothelial cells during the pathological process [94,95]. Unfortunately, the authors of those studies did not explicitly state the direct cause of the inhibitory effect of CMCS on angiogenesis. However, one may speculate that the blocking of venous endothelial cells might be the mechanism responsible for this effect, although other factors may also play a role. Another important factor in angiogenesis is the vascular endothelial growth factor (VEGF), which is a regulator of this process during growth and development, an inhibitor of metalloproteinase (TIMP-1) known for inhibiting neovascularization [96,97]. Research shows that CMCS can inhibit the 2D and 3D migration of human umbilical vein endothelial cells (HUVECs) in vitro. Jiang et al. found that carboxymethyl chitosan significantly decreased cell migration compared with a control group, which was based on two assays—the wound migration assay and the transwell migration assay. Moreover, the biomaterial inhibited tumor growth and CD34 (which is an early biomarker of bone marrow regeneration in the course of chemotherapy for acute leukemia) expression in hepatocarcinoma 22 tissues in vivo. Mice serum levels of TIMP-1 and VEGF might be controlled by CMCS, which highlights its anti-tumor action resulting from its anti-angiogenesis function [92]. CMCS was demonstrated to downregulate the expression of basic fibroblast growth factor (bFGF), which is one of the key pro-angiogenic factors. Downregulating this growth factor can inhibit the signaling pathways that promote angiogenesis. However, this will be discussed in depth in a section on the fibroblast effect [92]. A study carried out by Dai et al. used carboxymethyl chitosan–graft-poly(ε-caprolactone) copolymers (CMCS-g-PCL), which were synthesized through ε-caprolactone’s ring-opening polymerization from CMCS’s hydroxyl groups. Methanesulfonic acid was utilized as a catalyst and solvent during the five-hour reaction conducted at 45 °C in an argon environment. Then, the copolymer was used to encapsulate apatinib. Their results indicated that hydrogel micelles have an inhibiting effect on HUVEC cells, as the IC50 was ca. 3.1 μg/mL, suggesting their effective drug delivery function for anti-angiogenesis cancer therapy [98]. Another study conducted by Ya et al. utilized carboxymethyl chitosan cross-linked with activated γ-polyglutamic acid, which formed an injectable hydrogel that effectively killed tumor cells and bacteria under 808 nm laser irradiation. The hydrogel also exhibited antibacterial properties and anti-tumor efficiencies, which may be attributed to CMCS anti-angiogenesis properties [76].

It is also worth mentioning that CMCS-based biomaterials can be designed in a way to promote angiogenesis and vascular regeneration, which highlights the versatility of this polysaccharide derivative [93]. CMCS can be loaded with bioactive glass or magnesium ions, enabling the stimulation of expression of VEGF and bFGF mentioned earlier. This results in the promotion of angiogenesis and vascular regeneration. One study utilized carboxymethyl chitosan hydrogel for healing purposes in partial-thickness burn wounds. The developed CMCS hydrogel, hyaluronic acid (HA), and silver (Ag) showed significant wound contraction, granulation tissue formation, inflammatory infiltration, and collagen fiber deposition in the in vivo studies with rat animal models. The CMCS/HA/Ag association was found to be the most effective in the initial healing phase, while the CMCS/HA compositions were the best in subsequent phases. When focusing on angiogenesis, the process was more effective in the experimental group than in the control treated with physiologic solution and hyaludermin, which was caused by collagen fibers deposition, as further mentioned in the paper [23]. Another study aimed to develop a multifunctional hydrogel dressing for infected wounds. The hydrogel, composed of quaternized chitosan, methacrylate anhydride-modified collagen, and oxidized dextran, was cross-linked to improve stability and incorporate silver nanoparticles in situ. The hydrogel was evaluated for its biocompatibility, antibacterial properties, and wound-healing efficacy in an animal model. Zhao et al. also showed that a chitosan-based multifunctional hydrogel reduced silver ions to silver nanoparticles and exhibited good biocompatibility and antibacterial properties. More importantly, the promotion of the formation of epithelia and blood vessels was also observed, making it a potential clinical infected wound dressing application [99]. As previously mentioned, bioactive glass may be a valuable additive for wound healing, not only when it comes to chitosan-based hydrogels. Mehrabi et al. developed a hydrogel based on thiolated chitosan/carboxymethyl cellulose, which contained borate bioactive glass. The experiment was conducted using a mouse animal model and showed evident wound healing. Ultimately, they observed constant angiogenesis, remodeling, and quicker wound healing, all of which suggested that such hydrogels can be advantageous for the restoration of skin abnormalities [100]. Yang et al. also used a carboxymethyl chitosan-based hydrogel for wound healing. They used *Bletilla striata* polysaccharide (BSP) sponges for initial hemostasis and CMCS hydrogels loaded with panax notoginseng saponines (PNS) for angiogenesis. The proposed asymmetric double-layer dressing (BSP-PNS-CMCS) combined hemostasis and angiogenesis for wound healing in hemorrhagic wounds. In vivo experiments showed that the dressing reduced bleeding, accelerated wound closure, promoted angiogenesis, and accelerated collagen deposition in infected skin wounds, making the CMCS-containing material a good candidate for wound healing [101]. The versatility of CMCS and its hydrogels in modulating angiogenesis, either by inhibiting it in cancer or promoting it in wound healing and tissue engineering, makes them highly promising biomaterials for various biomedical applications. The ability to fine-tune the angiogenic response by incorporating different additives or combining CMCS with other materials further enhances their potential in the field of biomedicine.

### 4.2. Mechanism Underlying the Antibacterial Properties

The broad-spectrum antibacterial properties of CMCS and its hydrogels are attributed to bacterial cell membrane disruption, the inhibition of bacterial growth and proliferation, synergistic effects with other antimicrobials, the presence of ionogenic groups, pH sensitivity, and molecular weight dependence [21,89].

First, carboxymethyl chitosan hydrogels have positively charged amino groups that enable the interaction with negatively charged bacterial cell membranes. This may lead to disrupting the integrity of said membranes, resulting in cell death. CMCS macromolecules can also enter the bacterial cell and interfere with various processes like inhibiting enzyme activity or DNA synthesis [89,102]. Another aspect is metal ion chelation, which can be performed by CMCS. When metal ions essential for bacterial survival undergo chelation, bacteria are deprived of nutrients, inhibiting their growth [89,103]. The hydrogel form is beneficial as well since this network can act as a physical barrier, preventing the unwanted attachment and proliferation of bacteria on the surface [103]. In addition, carboxymethyl chitosan-based hydrogels may be combined with typical antimicrobial agents of chemical compounds (e.g., chlorhexidine gluconate) and inorganic metal nanoparticles releasing ions. This allows the substances to be released in a controlled manner and enhances the antibacterial efficacy already present because of CMCS chemistry [21].

For example, one study aimed to synthesize nanocomposites of anisaldehyde and salicylaldehyde carboxymethyl chitosan (CMC)-Schiff bases/silver nanoparticles to have antibacterial activity against various Gram-positive and Gram-negative bacteria (*Bacillus subtilis*, *Staphylococcus aureus*, *Streptococcus faecalis*, *Escherichia coli*, *Neisseria gonorrhoeae* and *Pseudomonas aeruginos*). Their cytotoxic activity against liver carcinoma cells and breast cells were evaluated [104]. Another report also confirmed the antibacterial properties of chitosan-based hydrogels enriched with metal ions (e.g., Ag^+^, Cu^2+^, Zn^2+^). Those hydrogels decreased or maintained the minimal inhibitory concentration (MIC) as well as the minimal bactericidal concentration (MBC) of *S. aureus* and *E. coli*. The biggest decreases were observed in chitosan-based hydrogels with Ag^+^ of MIC and MBC from 16 to 0.5 and 1 for *S. aureus*, respectively, and for both parameters from 32 to 1 for *E. coli* [103]. Wahid et al. aimed to develop injectable and self-healing hydrogels based on carboxymethyl chitosan and zinc ions. The hydrogels were characterized by their mechanical properties, thixotropy, injectability, and antibacterial activity. Using the agar diffusion method, the antibacterial properties of the produced supramolecular hydrogels were investigated against *S. aureus* and *E. coli*. Such characteristics were attributed to zinc ions as well as the hydrogel itself; however, it is worth mentioning that mechanical properties of hydrogels were dependent on the concentration of Zn^2+^ [105]. On the other hand, Xu et al. examined CMCS and silver nanoparticles coated onto cotton fabric, which demonstrated excellent antibacterial properties and laundering durability. The coating showed over 95% reduction rates in *S. aureus* and *E. coli* after 50 laundering cycles, offering potential applications in sportswear, socks, and medical textiles [106]. Yan et al. developed a hydrogel based on carboxymethyl chitosan and oxidized sodium alginate copolymerized with an acrylamide monomer. Through the use of composite hydrogel, wound healing in a model of full-thickness wounds infected with *S. aureus* was significantly accelerated without resulting in scarring. In general, their work provided new insight into the development of multipurpose hydrogel dressings for the treatment of wound infections [107].

### 4.3. Induction of the Anti-Inflammatory Effect

It has been demonstrated that carboxymethyl chitosan and its hydrogels possess anti-inflammatory potential that can aid wound healing and lessen inflammation in wounds and around medical implants or prostheses [108].

There are several mechanisms involved that allow CMCS to stimulate anti-inflammatory effects. The key ones are the modulation of inflammatory cytokines, the inhibition of inflammatory cell infiltration, and increasing anti-inflammatory mediators as well as potential antioxidant effects [89]. Carboxymethyl chitosan has been found to decrease pro-inflammatory cytokines such as IL-1β, IL-6, and TNF-α while being able to increase the expression of anti-inflammatory cytokine IL-10 [87,109,110]. Its hydrogels are also capable of reducing the infiltration of inflammatory cells, for example, neutrophils into the wound site, which results in lowering the inflammatory response. Increasing anti-inflammatory mediators such as osteoprotegrenin (OPG) combined with decreasing molecules like metalloproteinases (MMPs) also shifts the environment to an anti-inflammatory state [108]. Another advantage of CMCS is its ability to release antioxidant compounds like NPs of _h_CeO_2_ doped with Cu_5.4_O, which can scavenge reactive oxygen species (ROS), thus reducing oxidative stress and promoting an anti-inflammatory environment [87]. Gao et al. proved the increase in IL-10 in a mouse model with ameliorates hepatic fibrosis. Histologic examination and immunohistochemical examination demonstrated that CMCS was successful in reducing the host immune response generated by adenovirus (Ad). The authors showed that the development of hepatic fibrosis was effectively inhibited by repeated intrahepatic injections of Ad-IL10 combined with CMCS. All these findings point to the possibility that CMCS can enhance the potential of Ad-mediated gene therapy by reducing the host immune response and permitting re-administration and long-term transgene expression [88]. In a rabbit knee replacement model, carboxymethyl chitosan enhanced osteogenesis and decreased inflammation. CMC-treated rabbits were compared to controls for bone deposition, inflammation, and osteoblast/osteoclast markers. CMCS promoted cell proliferation and bone formation and increased their viability. It reduced inflammation around prostheses and promoted bone deposition. The CMCS-treated group had higher osteocalcin content and an increased OPG/RANKL ratio, although the exact numerical value for osteocalcin content was not provided in the excerpts. This suggested CMCS can inhibit inflammatory responses and promote osteogenesis [108]. Sun et al. confirmed that non-scar wound healing is improved by carboxymethyl chitosan nanoparticles loaded with the bioactive peptide OH-CATH30. CMCS-OH30 NPs were prepared using an ionic gelation method, allowing for the encapsulation of the bioactive peptide OH30 within the CMCS matrix. CMCS also has antibacterial properties, helping prevent infection in wounds. The combination of CMCS and OH30 enhanced antimicrobial efficacy against pathogens like *Pseudomonas aeruginosa*. CMCS-OH30 NPs also regulated cytokine expression, promoting a more favorable healing process. The positive surface charge of CMCS-OH30 NPs facilitated ionic interactions with negatively charged cell membranes, enhancing cellular uptake and bioadhesion. The study found that CMCS-OH30 NPs, a biodegradable drug delivery system, significantly accelerated wound healing and showed better prognosis because of the OH30 released from the nanoparticles. The system caused enhanced cell migration and granulation tissue formation while downregulating pro-inflammatory cytokine expression by at least half of the initial values [111]. Carboxymethyl chitosan was also used to improve ulcerative colitis (UC) treatment. Zhang et al. loaded CMCS with modified astaxanthin (AXT) nanoparticles (CMCS-AXT-NPs). In vitro research showed that CMCS-AXT-NPs had better antioxidant and anti-inflammatory properties. Moreover, dextran sulfate sodium salt (DSS)-induced colitis clinical symptoms were relieved by CMCS-AXT-NPs, including fecal hemorrhage, colon shortening inhibition, and body weight maintenance. Most importantly, DSS-induced oxidative damage was lessened by CMCS-AXT-NPs, which also inhibited the expression of pro-inflammatory cytokines such as TNF-α, IL-6, and IL-1β. The levels of TNF-α, IL-6, and IL-1β were reduced by 86.4%, 68.4%, and 81.9%, respectively, compared with the control group [112]. Another study focused on CMCS/plantamajoside hydrogel for burn wounds. The expressions of IL-1β, IL-6, and TNF-α were considerably reduced (60%, 50%, and 55%, respectively) by the combined hydrogel, while IL-10 expression was elevated (70%), compared with the control group consisting of subjects not treated with the hydrogel. Yu et al.’s findings also included increased angiogenesis and collagen deposition. Their results suggested that by decreasing the inflammatory response and accelerating angiogenesis and collagen deposition, the CMCS-containing hydrogel facilitated the healing of burn wounds [113]. A bioactive CMCS hydrogel cross-linked with natural phenolics was synthesized using the enzymatic method with *Myceliophthora thermophila laccase*. The hydrogels demonstrated excellent antioxidant and anti-inflammatory properties, with a 4-fold higher free radical scavenging activity than without phenolics. Furthermore, the hydrogels exhibited an anti-inflammatory impact, as demonstrated by the suppression of the overexpressed enzymes in chronic wounds, myeloperoxidase (MPO), matrix-metalloproteinase-1 (MMP-1), and human neutrophil elastase (HNE). With the highest reaching over 90%, achieved by 5 mg of a sinapyl alcohol–CMCS hydrogel [114]. The anti-inflammatory potential of CMCS and its hydrogels, mediated through various mechanisms, makes them highly promising materials for a wide range of biomedical applications, particularly in wound healing and reducing inflammation around medical implants or prostheses. The ability to modulate inflammatory cytokines, inhibit inflammatory cell infiltration, increase anti-inflammatory mediators, and provide antioxidant effects further enhances the therapeutic potential of these biomaterials.

### 4.4. Effect on Fibroblasts

Fibroblasts are spindle-shaped cells present in most tissues and organs that interact with extracellular matrix components [115]. They are differentiated mesenchymal cells that play a role in tissue homeostasis and diseases by generating complex extracellular matrix and establishing signaling niches via biophysical and biochemical inputs [116].

CMCS have been shown to be non-cytotoxic to fibroblasts, which is a critical prerequisite for the majority of materials employed in biomedical applications. The Live/Dead test performed on human fibroblasts by Kłosiński et al. demonstrated that CMCS hydrogels, manufactured with the radiation method in the presence of PEGDA cross-linker, did not cause cell death and, in some compositions, substantially improved cell viability [26]. Additionally, in an experiment carried out by Yu et al., CMCS/plantamajoside, a natural Chinese herbal medicine with biological activity and antioxidant properties and the ability to reduce inflammation and promote wound healing, hydrogels showed an increase in viability (about 60%) compared with a control group, along with increased angiogenesis (about 70%) and collagen deposition (about 80%) and a decreased inflammatory response of L929 fibroblast—the inflammatory markers were reduced by approximately 50% [113]. Fibroblast proliferation is strongly impacted by the composition of CMCS hydrogels. For example, Chen and colleagues showed that CMCS increased the proliferation of normal skin fibroblasts considerably compared with a control group—keloid fibroblasts cultured without additives—with increases reaching up to 130%. However, it prevented the multiplication of keloid fibroblasts, decreasing their numbers to 78 compared with the control group, which caused excessive scar tissue formation. This selective proliferation is useful in regulating and adjusting tissue development in therapeutic settings [117]. Hao et al. conducted animal experiments and found that bio-multifunctional benzaldehyde-terminated 4-arm PEG (4-arm-PEG-CHO)/carboxymethyl chitosan containing fibroblast growth factors prepared using the Schiff base reaction enhanced full-thickness diabetic wound healing. This was achieved by boosting epithelialization, collagen synthesis, hair follicle development, and the formation of new blood vessels. In the Schiff base reaction, the primary amine reacts with an aldehyde to form an imine (Schiff base). This reaction can occur under mild conditions, which is beneficial for in situ applications. The imine bonds formed are reversible, allowing for self-healing properties and adaptability in response to environmental stimuli, such as pH changes; the formulation is also easily injectable [118,119].

Another factor impacted by CMCS in fibroblasts is cell migration. A review by Szulc and Lewandowska suggested that CMCS shows enhanced cell migration and proliferation compared with standard chitosan hydrogels [120]. As previously mentioned, in Yu et al., hydrogels formulated with plantamajoside showed substantial cell migration, with the greatest rate being an approximately 80% increase compared with the control group reported in the highest concentration group. This suggests that CMCS hydrogels can efficiently assist in bringing fibroblasts to sites of tissue damage, hence encouraging the healing processes. The authors showed that CMCS hydrogels suppressed pro-inflammatory cytokines such as IL-1β, IL-6, and TNF-α, which subsequently accelerated the healing processes. This reduction in inflammation can help create a better environment for wound healing, allowing fibroblasts to work more efficiently throughout the repair process [113]. In addition to the previously mentioned processes, CMCS hydrogels have been shown to regulate the expression of some genes related to inflammation and healing in fibroblasts. Wang and coworkers demonstrated that a CMCS/sodium alginate composite hydrogel loaded with the mVEGF165/TGF-β_1_ gene can boost VEGF and TGF-β_1_ expression related to inflammation and healing in fibroblasts, which is necessary for angiogenesis and revascularization during wound healing. As demonstrated by the Elisa method, VEGF and TGF-β1 were expressed in NIH3T3 cells after in vitro transfection. Transfection efficiency increased with release time, reaching 3–4 times greater levels by day 9 compared with day 1. VEGF and TGF-β1 stimulate the formation of new blood vessels, which aids in the delivery of oxygen and nutrients to the wound site, thus accelerating the healing process [121]. A carboxymethyl chitosan hydrogel filled with basic fibroblast growth factor (bFGF) demonstrated increased fibroblast gene expression, boosting antiapoptosis and proangiogenesis simultaneously, which helped to reduce the fibrotic area caused by myocardial infarction, as demonstrated by histological examination [122]. As highlighted in the above examples, CMCS hydrogels are nontoxic to fibroblasts and can have beneficial effects on them and their functions. Fibroblast proliferation is heavily influenced by the composition of CMCS hydrogels. They can reduce inflammation, which allows fibroblasts to work with enhanced efficiency at the wound site. Moreover, they can also assist in delivering fibroblasts to the site, which also accelerates the healing process.

### 4.5. Collagen Deposition

Collagen deposition refers to the process in which collagen, an abundant extravascular protein, is laid down and accumulates in tissues. It is an important aspect of wound healing and tissue regeneration [123]. Hydrogels that incorporate collagen (usually type I collagen) are particularly promising for application in wound healing and skin regeneration [124,125].

As previously mentioned, the study carried out by Yu et al. found that CMCS hydrogels significantly enhanced collagen deposition in burn wound healing. This can be attributed to CMCS’s potential for stimulating the migration, proliferation, and production of cytokines by fibroblasts, which are essential cells in the healing process [113]. In a study by Patil et al., the researchers used an additionally oxygenated material of methacrylamide chitosan with perfluorocarbon chains (MACF) to develop hydrogel dressings for treating skin wounds. The effectiveness of the dressings in promoting wound healing was supported by evidence of increased collagen synthesis, which was one of the key indicators of faster healing, compared with the control groups. Masson’s trichrome histology was utilized to confirm collagen production directly in wound tissue parts, and collagen quantities were measured using image quantification. There was an increase in collagen area by two-and-a-half-fold in MACF as compared with a control group [126]. Zhou et al. created a CMCS–tannic acid biocompatibile composite hydrogel with remarkable mechanical properties by altering the concentration of tannic acid; as shown in their in vivo study, it was able to promote collagen deposition. This was confirmed again later in an in vivo wound healing study, which showed its potential in accelerating skin wound repair [127]. A study conducted by Gonçalves and coworkers investigated CMCS hyaluronic acid hydrogels with silver to improve collagen deposition in an experimental partial-thickness burn wound healing by increasing the abundance of collagen fibers. The group treated with CMCS and hyaluronic acid had more collagen than the others, e.g., about 15 to 5 compared with only CMCS [23]. The mechanism underlying CMCS hydrogels’ promotion of collagen deposition is complex and includes several cellular and molecular processes influencing cell viability, proliferation, and gene expression [10,113]. In addition, CMCS hydrogels were claimed to induce the production of growth factors, including fibroblast growth factor (FGF). FGF regulates collagen synthesis and plays a role in the healing processes. The presence of FGF in CMCS hydrogels increases collagen deposition by stimulating the proliferation and differentiation of fibroblasts, which are responsible for collagen production [128]. Lastly, CMCS hydrogels have been proven to control the inflammatory response, which is an important part of the healing process. CMCS hydrogels generate a favorable environment for collagen deposition by minimizing inflammatory infiltration and increasing the formation of granulation tissue [10]. Considering this with the aforementioned characteristics of these hydrogels, such as the stimulation of migration and proliferation of fibroblasts, crucial in the production of collagen, or the fact that when modified, they can enhance the synthesis of collagen itself, clearly shows that CMSC hydrogels impact collagen deposition and other related processes. This contributes to the high potential of CMCS and its hydrogels in wound healing and tissue regeneration applications. The mechanism of action of CMCS hydrogels is summarized in Table 2.

### 4.6. Summary of the Cellular Mechanisms of Action of Carboxymethyl Chitosan Hydrogels

CMCS hydrogels have the double ability to regulate angiogenesis. In order to exploit their dual capability, CMCS hydrogels may be customized to meet particular therapeutic requirements, making them useful biomaterials for a wide range of biomedical applications, including tissue engineering (mainly scaffolds), wound healing, and cancer treatment (Table 2). However, it is important to note that despite their advantageous characteristics and high potential for various medical applications, there are no products comprising CMCS on the market or in clinical trials. By reducing pro-angiogenic factors and inhibiting endothelial cell migration, they can prevent angiogenesis, which is advantageous in the treatment of cancer. On the other hand, under different circumstances, by promoting the production of growth factors like VEGF and bFGF, they can also aid in enhancing angiogenesis, which is essential for tissue engineering and wound healing. CMCS hydrogels’ antibacterial qualities are explained by a number of factors. These include metal ion chelation, interfering with intracellular processes, and breaking down bacterial cell membranes through electrostatic interactions. Furthermore, the hydrogel structure serves as a physical barrier to prevent bacterial colonization. It has been demonstrated that these systems work well against a variety of pathogens, including *S. aureus* and *E. coli*. Another important mechanism of CMCS hydrogels is their anti-inflammatory properties. The regulation of inflammatory cytokines is a crucial process. It has been demonstrated that CMCS increases the production of anti-inflammatory cytokines, like IL-10, while decreasing pro-inflammatory cytokines, like TNF-α, IL-1β, and IL-6, thus reducing the migration of inflammatory cells and increasing anti-inflammatory mediators such as osteoprotegerin (OPG). The anti-inflammatory benefits of CMCS are partly attributed to its antioxidant capabilities. Certain CMCS hydrogels have the ability to release antioxidant molecules, scavenging reactive oxygen species (ROS) and, therefore, mitigating oxidative stress and fostering an anti-inflammatory environment. Because of their anti-inflammatory properties, CMCS hydrogels are especially helpful in treating inflammatory diseases including ulcerative colitis, mending wounds, and reducing inflammation surrounding medical implants. Fibroblasts, which are essential cells in wound healing and tissue repair, are greatly impacted by CMCS hydrogels. The vitality and proliferation of fibroblasts is one of the main impacts. According to studies, CMCS hydrogels can increase the vitality of fibroblasts and are not harmful to them. Fibroblast proliferation is impacted by CMCS, which improves the migration, proliferation, and viability of fibroblasts. Notably, they have the ability to stimulate the growth of normal fibroblasts specifically while preventing the formation of keloid fibroblasts. Moreover, CMCS hydrogels control fibroblast gene expression, assisting the synthesis of cytokines and growth factors that are vital for wound healing. CMCS can also have a significant impact on collagen deposition. Through a number of ways, CMCS hydrogels propagate the deposition of collagen and increase collagen synthesis by promoting fibroblast activity. Additionally, they have the direct ability to affect fibroblasts’ expression of genes involved in collagen production and stimulate the production of growth factors that control the synthesis of collagen. Enhancing collagen deposition with CMCS hydrogels leads to superior wound healing results, such as quicker wound closure, stronger tissue, and less scarring. Because of this, CMCS hydrogels are very useful in applications like scaffolds for tissue engineering, regenerative medicine, and wound dressings. The properties and cellular effects of CMCS hydrogels are summarized in Table 3.

## 5. Summary

This review addresses a number of topics related to carboxymethyl chitosan hydrogels, including their synthesis, biochemical characteristics, biological uses, and roles in numerous cell processes. CMCS is typically obtained from chitosan via a reaction with chloroacetic acid, which substitutes carboxymethyl groups to amino and hydroxyl groups [45]. The mechanical performance of CMCS hydrogels can be tuned for particular applications. In principle, CMCS hydrogels possess the biological activity of their parent polysaccharide, chitosan, related to molecular weight and degree of deacetylation; thus, the presence of an ionogenic amine group. Nevertheless, they are distinct from chitosan because of stimuli-responsive swelling, where transitions occur at two pH values. This results in swelling at low pH, deswelling at a pH in the range of ca. 3 to 5, and extensive swelling at higher pH values, including physiological values. This is related to its amphoteric character, where amine groups are protonated at acidic pH and carboxylic groups deprotonated at pH neutral and above [129]. The characteristics of deswelling are due to both ionogenic groups being ionized; therefore, electrostatic attraction contracts the gel. Moreover, chitosan can be dissolved only in an acidic solution, and the amine will be protonated, whereas the CMCS linear polymer dissolves in water and aqueous solutions also at neutral pH [21,26].

The versatile biochemical traits represented by carboxymethyl chitosan hydrogels provide an extensive number of possible applications across different industry branches, with the main focus on biomedicine. Characteristics such as biocompatibility and biodegradability, demonstrated by a number of scientific groups, combined with tuned physicochemical properties and performance predestine them to be utilized for wound healing and tissue regeneration [10,23,61]. On the other hand, because of carboxymethyl modification, CMCS hydrogels manifest good elasticity, enhanced liquid absorption, tailored porosity, and modified mechanical strength, which also can be obtained and enhanced by incorporating other macromolecules or cross-linkers to form composite hydrogels, e.g., polyvinyl alcohol, poly(ethylene glycol) diglycidyl, and silane Ca^2+^. CMCS hydrogels can be also designated for the controlled release and delivery of various components, especially nanoparticles loaded with drugs, proteins, vitamins, and natural components, e.g., curcumin [21]. The presence of amino groups and antibacterial materials (chlorhexidine gluconate, silver, zinc, and copper nanoparticles) allows for the application of CMCS to combat diseases caused by bacteria, fungi, and microbes, e.g., *E. coli*, *S. aureus*, *Cutibacterium acne*, *Pediococcus pentosaceus*, and some types of *Candida* fungi (*C. albicans*, *C. krusei*, and *C. glabrata*). The antimicrobial activity, via disrupting the targeted bacteria’s DNA synthesis, metal ion chelation, and enzyme activity preventing the microbe from growing and surviving, makes CMCS hydrogels useful in wound healing, endoscopic submucosal dissection, anti-tumor recurrence applications, osteomyelitis, and mucosal and deep-tissue infection treatment applications.

Given the relationship between cellular mechanisms and CMCS hydrogels, the effects on angiogenesis, inflammation, fibroblasts, collagen, and antibacterial activities were discussed. Angiogenesis can be impacted by two diverse effects, i.e., inhibiting and promoting vessel and capillary development. Firstly, the inhibitory effect on angiogenesis can be essential for preventing the growth and spread of tumors in cancer patients, as well as for controlling the expression of pro-angiogenic factors [92]. These properties were exploited in research efforts featuring numerous drugs and compounds [21]. Secondly, by promoting angiogenesis, CMCS hydrogels, combined with, e.g., bioactive glass or magnesium ions, hyaluronic acid, and silver, can be applied for vascular regeneration, the restoration of skin abnormalities, burn wounds, and hemorrhagic wound healing [130]. CMCS hydrogel can participate in reducing inflammation states by inflammatory cytokine modulation (IL-1β, IL-6, or TNF-α), inflammatory cell infiltration inhibition, inflammatory mediator enhancement, and possible antioxidant effects. Therefore, it is hoped that they can aid the treatment of hepatic fibrosis and ulcerative colitis, wound healing, and osteogenesis promotion [88,108,111,113]. Regarding the effect of CMCS hydrogels on fibroblasts, they can enhance the migration and proliferation of these cells, as well as stimulate the production of collagen and epithelium and regulate gene expression [113]. The presented features may influence wound healing and myocardial infarction [121,122]. CMCS hydrogels have a positive impact on collagen deposition, which is associated with the production of growth factors, fibroblast migration and growth, and cytokine production. The biomedical implication of this feature is that it can be utilized for wound healing, skin repair, and bone tissue engineering [130].

**Figure 4:**
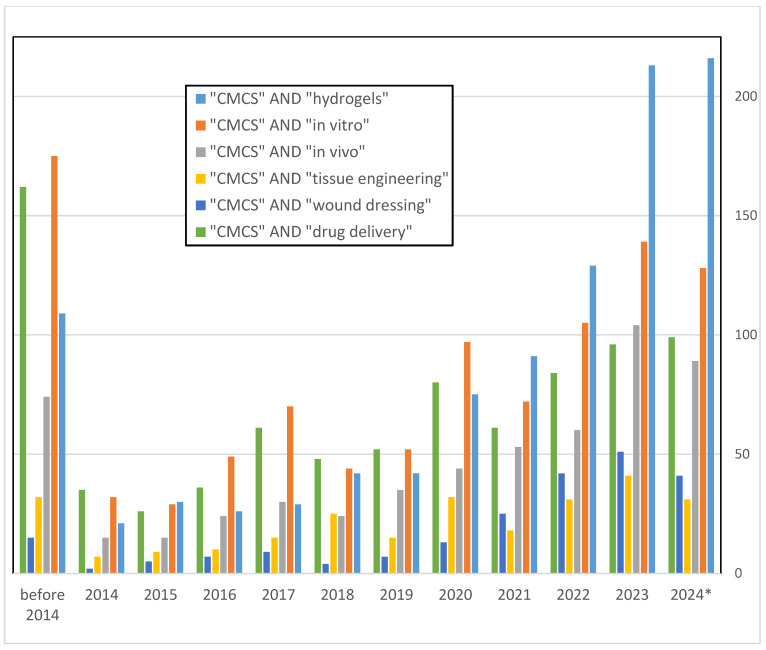
Number of publications on carboxymethyl chitosan indexed in the Scopus database, sourced from publication “title” or “abstract” or “keyword”. The number of publications in the last ten years* and summarized number of publications until 2014, encompassing “carboxymethyl chitosan” and “a specific keyword” of the most exploited hydrogel biomaterial application and biological testing (* in 2024, only 8 months were taken into account for the date of this publication).

The interest in carboxymethyl chitosan research cannot be disregarded, as the total number of publications encompassing CMCS indexed in Scopus is over 3600. Moreover, in the year of 2023 and 8 months of the year 2024, ca. 900 reports were published. This is an enormous increase because it is greater than the number of all publications on the CMCS subject published before the year 2014. More detailed statistics for the last ten years are presented in Figure 4.

Taking into account the biological and in vivo examinations in papers related to CMCS, the increase is 4.4 and 6,4 times, respectively, which is somewhat more than the general increase in CMCS interest of 3.4 times. Here, and henceforth, the increase is assumed as the ratio of the number of papers published in (2023 + 2024) to the number of papers published in (2014 + 2015). This implies that the studies on biomaterials with CMCS are in focus. Research on “carboxymethyl chitosan” combined with “hydrogels” increased by 8.4-fold, which is a direct indication of the importance of this subject. Interest in three specific biomedical applications increased as well. Namely, for tissue engineering, wound dressing, and drug delivery, the rise was 4.6 and more than 13 and 3.2 times, respectively. Drug delivery, the classical and fairly developed application of biomaterials, still captures the attention of scientist, as the general number of papers on this subject is high. A high increase in both tissue engineering and wound dressing applications, combined with CMCS, seems to be due to the low base ten years ago. Wound management, particularly of hard-to-heal injuries, is a major clinical challenge, and one has to admit that CMCS and hydrogels represent the most growing segment of scientific interest.

Concluding the addressed topic, carboxymethyl chitosan hydrogels are a valuable component for expanding the field of biomedicine and the management of several diseases as they demonstrate various highly desirable features. The hydrogels discussed in this article are materials that are worthy of further study and use. Based on the information provided, the future perspectives for CMCS hydrogels appear promising, with potential for further research and expanded applications in biomedicine, particularly in areas such as wound healing, tissue regeneration, drug delivery, and disease management, because of their versatile biochemical characteristics and beneficial cellular effects.

## Figures and Tables

**Table 1:** Comparison of CMCS hydrogel properties.

Property	Description	Potential Application
Biocompatibility [23,26,52,61]	Good biocompatibility No adverse effects on tissue Support cell viability and proliferation	Wound healing Tissue engineering Drug delivery
Mechanical properties [65,67,69,86]	Tailored solid-like characteristics Adjustable strength and elasticity Controlled by degree of carboxymethylation and cross-linking	Wound dressings Tissue scaffolds Controlled release systems
Controlled release [70,71,72,73]	High viscosity and gel-forming ability pH-responsive swelling Ability to encapsulate and release various compounds	Drug delivery Sustained release of proteins, vitamins, and other bioactive compounds
Antimicrobial efficacy [74,78,80,85]	Broad-spectrum antibacterial properties Effective against bacteria and fungi Can be enhanced with additives (e.g., silver nanoparticles)	Wound dressings Antibacterial coatings Infection prevention
Anti-inflammatory effect [87,88,89]	Modulation in inflammatory cytokines Reduction of inflammatory cell infiltration Potential antioxidant effects	Treatment of inflammatory conditions Wound healing Tissue regeneration

**Table 2:** Studies on mechanisms of action of CMCS hydrogels.

Effect	Mechanism of Action	Key Findings
Angiogenesis inhibition [92]	Endothelial cell migration inhibition Downregulation of pro-angiogenic factors	CMCS inhibited 2D and 3D migration of HUVECs, decreased CD34 expression in hepatocarcinoma tissues, and controlled serum levels of TIMP-1 and VEGF
Angiogenesis promotion [93]	Stimulation of growth factors	CMCS loaded with bioactive glass or magnesium ions stimulated the expression of VEGF and bFGF
Antibacterial [106]	Bacterial membrane disruption	CMCS hydrogels with silver nanoparticles showed >95% reduced rates against *S. aureus* and *E. coli* after 50 laundering cycles
Osteogenesis promotion [108]	Regulation of bone-related proteins	CMCS increased osteocalcin content and the OPG/RANKL ratio in a rabbit knee replacement model
Controlled drug release [73]	pH-responsive swelling	CMCS/starch hydrogels with CuO nanoparticles showed pH-dependent swelling and controlled release of amoxicillin
Collagen deposition [23]	Stimulation of fibroblast activity	CMCS/HA/Ag hydrogels promoted collagen fiber deposition in partial-thickness burn wounds
Anti-fungal activity [85]	Fungal growth inhibition	CMCS delayed growth of *C. albicans*, *C. krusei*, and *C. glabrata*
Gene therapy enhancement [88]	Reduction in host immune response	CMCS allowed for repeated administration and long-term transgene expression of IL-10 in a hepatic fibrosis model
Anti-inflammatory [87,108,109,110]	Cytokine modulation Antioxidant effect	CMCS hydrogels suppressed pro-inflammatory cytokines, increased anti-inflammatory mediators, and scavenged reactive oxygen species
Wound healing [113]	Promotion of fibroblast activity Cytokine modulation	CMCS/plantamajoside hydrogel increased cell viability, migration, and collagen deposition, reduced expression of IL-1β, IL-6, and TNF-α, and increased IL-10 expression

**Table 3:** Properties and cellular effects of carboxymethyl chitosan-based hydrogels for biomedical applications.

Hydrogel Composition	Cellular Action	Added Substance	Potential Application
Carboxymethyl chitosan [92]	Inhibitory effect on angiogenesis	-	Represses tumor angiogenesis
Carboxymethyl chitosan–graft-poly(ε-caprolactone) copolymers (CMCS-g-PCL) [98]	Inhibitory effect on angiogenesis	poly(ε-caprolactone) copolymers Apatinib (encapsulated)	Effective drug delivery Anti-angiogenesis cancer therapy
Carboxymethyl chitosan/activated γ-polyglutamic acid [76]	Inhibitory effect on angiogenesis	Activated γ-polyglutamic acid	Antibacterial properties Anti-angiogenesis cancer therapy
Carboxymethyl chitosan/hyaluronic acid/silver [23]	Promotion of angiogenesis	Hyaluronic acid Silver	Initial healing phase
Double-cross-linked multifunctional hydrogel (coc hydrogel) based on quaternized chitosan, methacrylate anhydride-modified collagen, and oxidized dextran [99]	Promotion of angiogenesis	Silver nanoparticles Collagen Oxidized dextran	Wound dressing application
Thiolated chitosan/carboxymethyl cellulose [100]	Promotion of angiogenesis	Borate bioactive glass	Wound dressing application
Carboxymethyl chitosan [101]	Promotion of angiogenesis	Bletilla striata polysaccharide Panax notoginseng saponines	Wound healing in hemorrhagic wounds
Anisaldehyde and salicylaldehyde carboxymethyl chitosan-Schiff bases/silver nanoparticles [104]	Antibacterial properties against various Gram-positive and Gram-negative bacteria	Anisaledehyde Salicylaldehyde Silver nanoparticles	Infections caused by *B. Subtilis*, *S. aureus*, *S. faecalis*, *E. coli*, *N. gonorrhoeae*, and *P. aeruginos*
Porous chitosan-based hydrogels cross-linked with gelatin and metal ions [103]	Antibacterial properties	Gelatin (GEL) Formaldehyde Metallic salts (Ag^+^, Cu^2+^, and Zn^2+^)	Infections caused by *S. aureus* and *E. coli*
Injectable self-healing carboxymethyl chitosan (CMCh) supramolecular hydrogels cross-linked by zinc ions (Zn^2+^) [105]	Antibacterial properties	Zinc ions	Infections caused by *S. aureus* and *E. coli*
Carboxymethyl chitosan and silver nanoparticles linked onto a cotton fabric surface [106]	Antibacterial properties Bacterial reduction in *S. aureus* and *E. coli*	Silver nanoparticles	Sportswear, socks, and medical textile
Carboxymethyl chitosan/oxidized sodium alginate/acrylamide monomer [107]	Antibacterial properties	Oxidized sodium alginate Acrylamide monomer	Treatment of wound infections
Carboxymethyl chitosan [88]	Anti-inflammatory effect Increase in IL-10	Adenoviral vector-IL10 mixed with CMC	Improvement in Ad-mediated gene therapy Hepatic fibrosis
Carboxymethyl chitosan [108]	Promotion of cell proliferation Decreased inflammation	-	Promotion of osteogenesis
Carboxymethyl chitosan [111]	Downregulation of the expression of several proinflammatory cytokines	Bioactive peptide OH-CATH30	Drug delivery system for non-scar wound healing
Carboxymethyl chitosan [112]	Antioxidant and anti-inflammatory properties	Modified astaxanthin nanoparticles	Ulcerative colitis treatment
Carboxymethyl chitosan/plantamajoside hydrogel [113]	Decreased the inflammatory response of L929 fibroblasts, increased angiogenesis, and increased collagen deposition Fibroblast cell migration	Plantamajoside	Healing of burn wounds
Phenolic-*O-*carboxymethyl chitosan hydrogels [114]	Antioxidant and anti-inflammatory properties	Laccase from Myceliophthora thermophile (e.g., catechol)	Chronic wounds treatment
Carboxymethyl chitosan/polyethylene glycol diacrylate [26]	Improved cell viability on human fibroblasts	Polyethylene glycol diacrylate	Wound healing
Carboxymethyl chitosan [117]	Increased the proliferation of skin fibroblasts and prevent multiplication of keloid fibroblasts	-	Regulation of tissue development in therapy
Bio-multifunctional benzaldehyde-terminated 4-arm PEG (4-arm-PEG-CHO)/carboxymethyl chitosan (CMCS)/basic fibroblast growth factor (bFGF) hydrogels (BP/CS-bFGF) [118]	Increased epithelialization, collagen synthesis, hair follicle development, and the formation of new blood vessels	Multifunctional benzaldehyde-terminated 4-arm Polyethylene glycol Basic fibroblast growth factor (bFGF)	Full-thickness diabetic wound healing
*N-*carboxymethyl chitosan/sodium alginate composite hydrogel [121]	Boosted inflammation and healing in fibroblasts gene expression	Sodium alginate mVEGF165/TGF-β1 gene-loaded	Burn wound treatment Skin regeneration
Pyridyl disulfide-modified carboxymethyl chitosan [122]	Increased fibroblast gene expression Anti-apoptosis properties Angiogenesis stimulation	Disulfate	Reduction in fibrotic area in myocardial infarction
Oxygenated methacrylamide chitosan with perfluorocarbon chains hydrogel [126]	Increased collagen synthesis	Methacrylic anhydride Pentadecafluorooctanoyl chloride (additionally oxygenated)	Wound healing
Carboxymethyl chitosan/tannic acid hydrogel [127]	Promotion of collagen deposition	Tannic acid	Wound healing
Carboxymethyl chitosan/hyaluronic acid/silver [23]	Promotion of collagen deposition	Hyaluronic acid Silver	Skin regeneration Burn wound healing
(−)-epigallocatechin-3-*O-*gallate-cross-linked carboxymethyl chitosan-based hydrogels [47]	Promotion of collagen deposition	(−)-epigallocatechin-3-*O-*gallate	Skin regeneration Burn wound healing

## Data Availability

No new data were created or analyzed in this study. Data sharing is not applicable to this article.
